# Anesthesia decision analysis using a cloud-based big data platform

**DOI:** 10.1186/s40001-024-01764-0

**Published:** 2024-03-25

**Authors:** Shuiting Zhang, Hui Li, Qiancheng Jing, Weiyun Shen, Wei Luo, Ruping Dai

**Affiliations:** 1 Department of Anesthesiology, The Second Xiangya Hospital, Central South University, Changsha, 410008 Hunan China; 2 Anesthesia Medical Research, Center Central, South University, Changsha, 410008 Hunan China; 3 Department of Otolaryngology Head and Neck Surgery, Hengyang Medical School, The Affiliated Changsha Central Hospital, University of South China, Changsha, 410000 Hunan China

**Keywords:** Anesthesia analysis, Decision-making, Big data, Cloud-based, Platform, Precision medicine

## Abstract

Big data technologies have proliferated since the dawn of the cloud-computing era. Traditional data storage, extraction, transformation, and analysis technologies have thus become unsuitable for the large volume, diversity, high processing speed, and low value density of big data in medical strategies, which require the development of novel big data application technologies. In this regard, we investigated the most recent big data platform breakthroughs in anesthesiology and designed an anesthesia decision model based on a cloud system for storing and analyzing massive amounts of data from anesthetic records. The presented Anesthesia Decision Analysis Platform performs distributed computing on medical records via several programming tools, and provides services such as keyword search, data filtering, and basic statistics to reduce inaccurate and subjective judgments by decision-makers. Importantly, it can potentially to improve anesthetic strategy and create individualized anesthesia decisions, lowering the likelihood of perioperative complications.

**Supplementary Information:**

The online version contains supplementary material available at 10.1186/s40001-024-01764-0.

## Introduction

As the digital era progresses, the amount and velocity of public health data are rapidly increasing. Big data analytical techniques, such as statistical analysis, data mining, machine learning, and deep learning, have developed significantly in recent years, attracting the attention of researchers and scientists in a wide range of applications [1–3]. Making decisions based on concrete evidence via a big data platform is crucial and has a significant impact on precision medicine and personalized therapy implementation [4–7]. The field of anesthesia poses challenges in terms of both technical competence and decision-making, the latter being frequently influenced by time constraints and continuously changing clinical conditions [8, 9]. However, typical medical decision-making is unstructured and haphazardly randomly utilizes evidence. Therefore, a more organized strategy based on decision analysis is required.

Focusing on these unserved needs and well-recognized problems, we reviewed the big data platforms in anesthesiology, investigate big data analysis in decision-making in anesthesia, and developed a cloud-based big data platform for anesthetic decision-making. First, we outline the important steps of the big data platform's design, such as raw data acquisition, classification, and normalization. Furthermore, support facilities for the big data platform are listed. To illustrate the process of this approach, we outline a method for its execution, determining a strategy for anesthesia. Moreover, we illustrate the potential of these innovations for more refined patient management during the perioperative period with examples of anesthesia decision modes of cloud-based big data platforms. Finally, based on big data, we discuss how anesthesiologists and data scientists should collaborate to capture data across the perioperative care period and provide clinical context to achieve the highest quality of anesthesia for patients.

In this study, the most recent advances in big data platforms for anesthesiology were reviewed, and then, a decision platform was designed for implementation in the near future.

## Big data platform in anesthesiology

The era of electronic health records, genomics, proteomics, and pathology data has boosted information technology resources, allowing the creation of big data platforms capable of swiftly delivering useful clinical evidence [10]. Medtronic Iberica® S.A., a Spanish company, recently established the SCOOP platform, a national-level scientific cloud-based big data solution for implantable cardioverter defibrillators [11]. Makkie et al. are developing a data management system to organize large-scale functional magnetic resonance imaging datasets that show the considerable performance benefits of their algorithms/methods for performing distributed dictionary learning [12]. The big data platform has ushered in a new era of better service delivery and clinical problem solving [13].

**Fig. 1:**
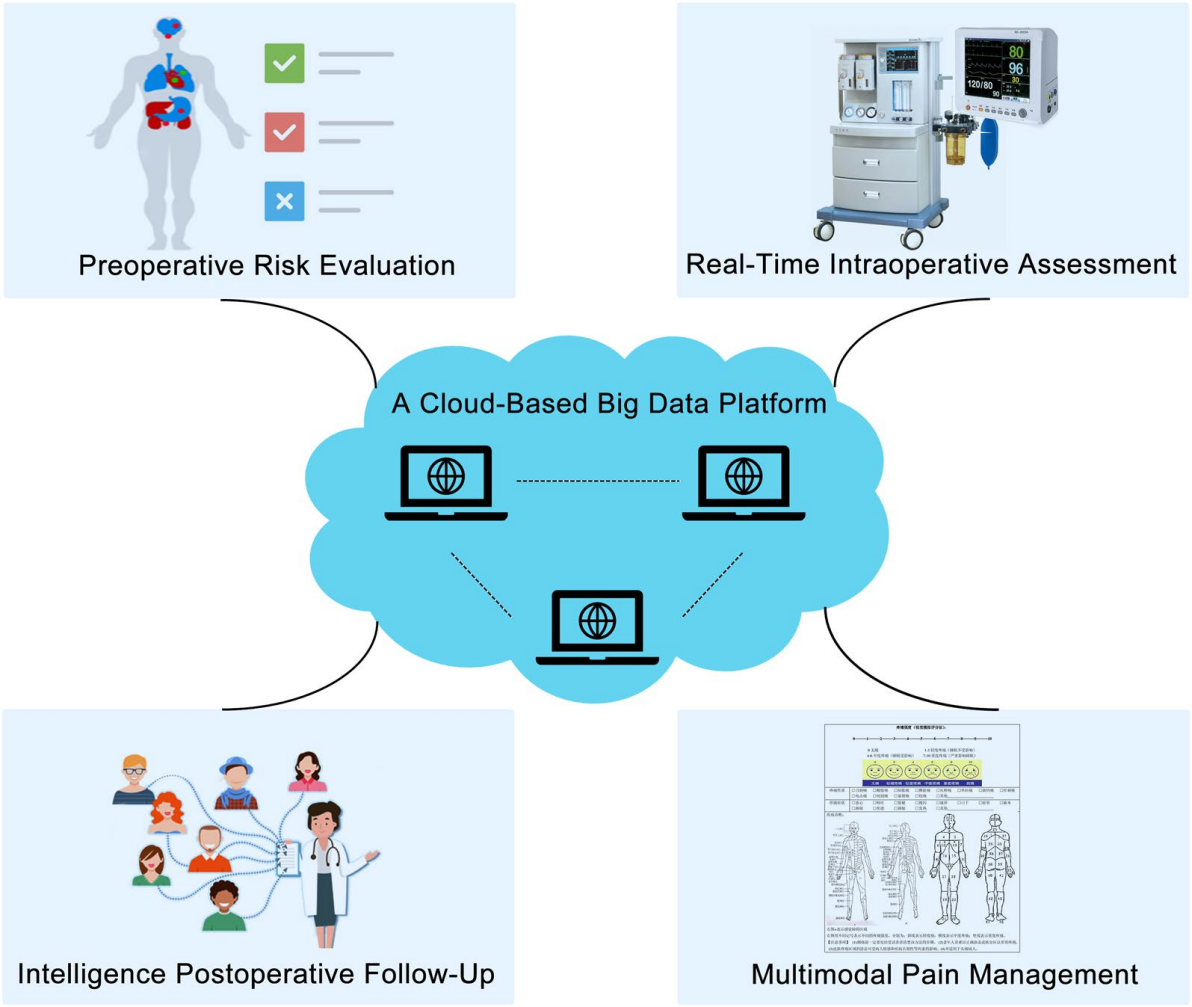
Big data platform in anesthesiology

With the accumulation of large amounts of perioperative period-related data in anesthesiology, big data analysis technology is gradually being applied to preoperative risk evaluation, real-time intraoperative assessment, intelligence postoperative follow-up, and multimodal pain management (Fig. 1). Poucke, et al. developed robust processes for automatic model building, parameter optimization, and evaluation. They applied scalable predictive analysis in critically ill patients using a visual open data analysis platform, and relied on visual tools for the extract, transform, and load (ETL) process on Hadoop, and predictive modeling in RapidMiner [14]. Klumpner et al. utilized various databases to assist the obstetric anesthesiology community better understand adverse maternal events associated with obstetric anesthesia [15]. A recent study demonstrated that patient assessments and provider reminders driven by clinical decision support systems integrated into anesthesia information management systems reduced postoperative nausea and vomiting and improved adherence to an institutional glucose management protocol [16, 17]. The evidence presented above demonstrated that big data platforms have optimized perioperative anesthesia decision-making and evaluation.

However, relatively few big data platforms have been developed in the field of anesthesia. Further, statistics on the specific anesthesia plan development and analysis platform, which include anesthesia procedure, anesthetic dosage, maintenance dosage, and postoperative recovery time, are, to some extent, inaccessible. In this section, we describe the design of such a platform to optimize the clinical anesthesia plan.

## Anesthesiology decision analysis platform

### Fundamental procedures of the anesthesiology decision analysis platform

**Fig. 2:**
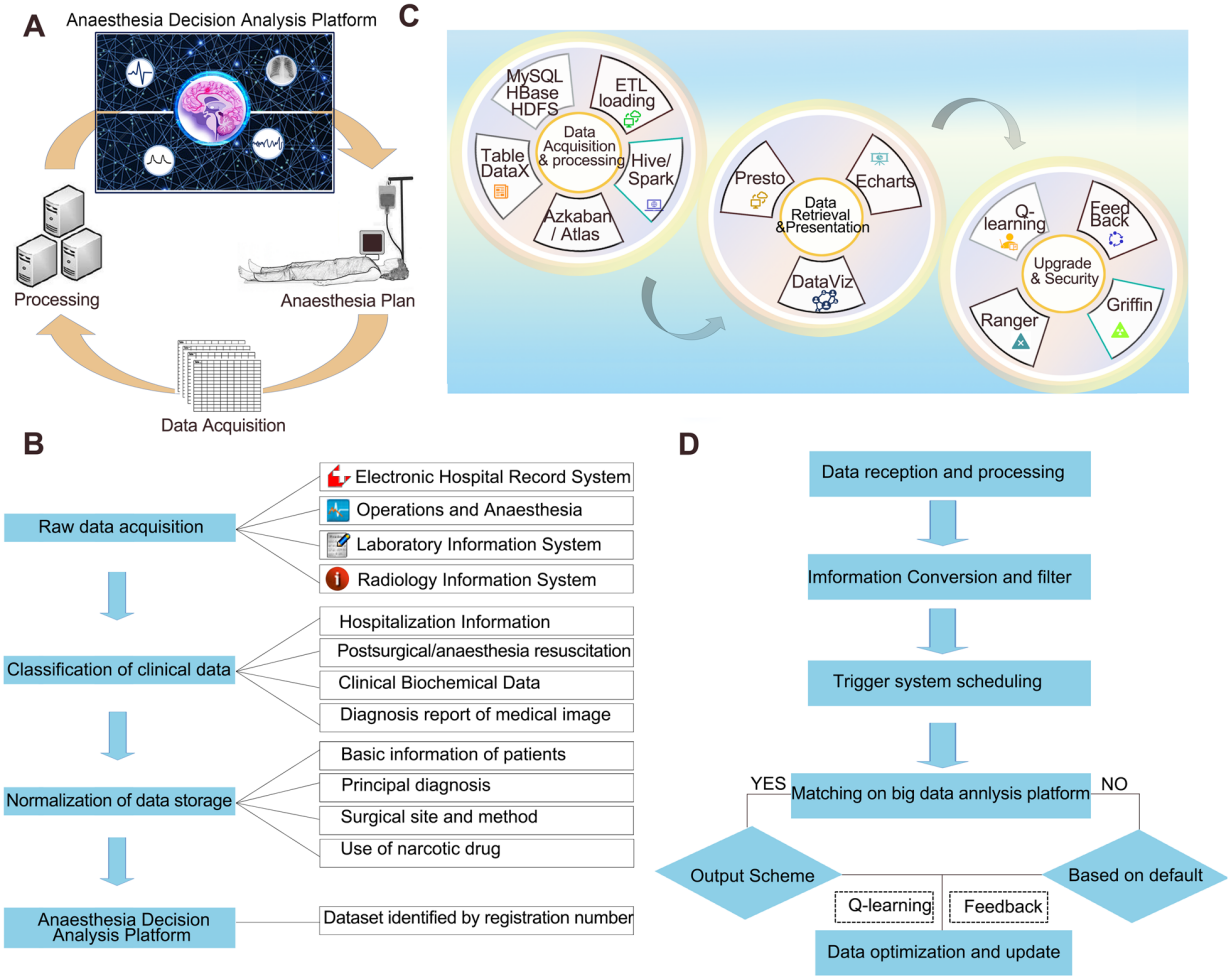
Design of the Anesthetic Decision Analysis Platform, involving the working loop (**A**), architecture (**B**), fundamental procedures (**C**), and output workflows (**D**)

We constructed an Anesthesiology Decision Analysis Platform (Fig. 2A) equipped with perioperative clinical anesthesia-related data input; some of the fundamental procedures are as follows (Fig. 2B):

i. raw data acquisition from medical systems, such as electronic hospital records, operations and anesthesia, laboratory information, and radiology information systems (the corresponding icon represents the Second Xiangya Hospital's associated medical system) (Table 1 lists the variables that need to be collected)
ii. clinical data classification into categories and intermediate outputs. Clinical data including hospitalization information, postsurgical/anesthesia resuscitation records, clinical and biochemical data, and medical image diagnosis report;
iii. normalization of data storage into major parts, such as basic patient information, principal diagnosis, surgical site and method, and narcotic drug use.
iv. Creation of a big data platform and dataset that can be identified using registration numbers.

**Table 1:** Variables included in construction of anesthesiology decision analysis platform

Feature type	Features	Preprocessing
*Patient characteristics*		
Continuous	Age, height, weight, BSA, BMI and Tumor size	Normalization
Categorical	Sex, Charlson Comorbidity Index, Mallampati grading, mouth opening, Reoperation, tumor stage and differentiation, Metastasis, functional capacity, ASA physical status, ASA, emergency status, anesthesia type, and surgery type	One-hot encoding
Categorical comorbid conditions	Hypertension, coronary artery disease, prior myocardial infarction, congestive heart failure, diastolic function, left ventricular ejection fraction, aortic stenosis, atrial fibrillation, prior stroke or transient ischemic attack, pacemaker or implanted defibrillator, peripheral artery disease, deep venous thrombosis, pulmonary embolism, diabetes, chronic kidney disease, ongoing dialysis, pulmonary hypertension, chronic obstructive pulmonary disease, asthma, obstructive sleep apnea, cirrhosis, any cancer, gastroesophageal reflux, anemia, positive Coombs test result, dementia, ever-smoker, and alcohol addiction	One-hot encoding if not binary

The Anesthesiology Decision Analysis Platform aims to create links between artificial intelligence and anesthesia choices (or decisions) for surgical patients. Consequently, the design objectives of the cloud-based big data platform are twofold: first, to improve clinical decision-making at the system level by better analyzing data collected dynamically from diverse medical system sources; and second, to improve the flow of raw data generated from clinical settings for big data research.

The study was approved by the Research Ethics Committee of Second Xiangya Hospital, Central South University, Changsha, China (LYF 20240022).

### Architecture of the anesthesiology decision analysis platform

The usage of large amounts of data requires the use of technological tools for data gathering from many sources and systems, as well as data transformation, storage, analysis, and visualization (Fig. 2C, Additional file 2: Table S1). Programming languages, such as C/C +  + , Python, Java, and Perl, are tools used to create purpose-built application programs through which instructions are issued to computers to achieve the desired objectives [18]. In addition, technological infrastructure for data storage is employed, which includes MySQL, HBase of Hadoop, and the Hadoop Distributed File System relational database system built on a Linux server [19]. Big data software, which facilitates the time-constrained processing of continuous information flows to produce actionable intelligence [20], is required for ‘data acquisition & processing’, ‘big data platform running’, and ‘system upgrade and platform security’.

#### Data acquisition & processing

Because medical records and information are now stored in disparate data formats, they are typically kept in comma-separated values (CSV) or table data format and can simply be stored on platforms utilizing these data formats [21]. The upload of data to the cloud is performed using DataX offline data synchronization software. Configuration environments for Python [22], Anaconda [23], and R language [24] are used for the analysis of intermediate output data. When data are uploaded into the cloud-based platform, the data collecting service begins ETL into a distributed database system [25]. After their upload into cloud storage, data are processed by a cloud-computing engine, such as Hive or Spark, distributed using the Azkaban System, and maintained by the Apache Atlas metadata management system [26].

#### Data retrieval and presentation

A data exploration portal (or simply a data portal) is employed for viewing, exploring, and downloading data from the platform, which uses the Presto data retrieval engine, a distributed open-source structured query language engine developed by Facebook’s Data Infrastructure Group. Apache Echarts [27] and DataViz [28] technology are used to meet the dynamic needs of visual interfaces and multidimensional visualization.

#### Upgrades & platform security

As the platform data accumulates, the Q-learning and Feedback algorithm is used to update and optimize parameters [29]. Furthermore, big data are said to be “new oil, but not clean oil,” an expression that seeks to warn that it can be both a critical driver of automation in the field of medicine and a source of information leakage. To maintain data storage security and compliance, the system employs Apache Ranger and Griffin software, which assures data disaster recovery capability and provides audit management and strategy analysis for the platform [30, 31].

### Workflow of anesthesiology decision analysis platform

Anesthesia decision-making platforms developed with anesthesia big data input may be knowledge-based, with a sophisticated approach to causality algorithms, or non-knowledge-based (Fig. 2D), essentially designed to provide cognitive aids to the anesthetist [32].

The user logs into the big data platform and preoperatively enters the registration number of the case. The system extracts essential parameters while also detecting anomalous variables, ensuring anesthetic risks and drug compliance, and checking big data. Simultaneously, system identification recognizes and processes the relevant parameters, followed by information conversion and filtering. The filtering rules include the column, equivalent rules (> , = , and), age, sex, height, weight, and diagnosis. The system also detects anomalous findings based on the risk prediction results and whether the patient's clinical parameters exceed the threshold. In the present scenario, the normalization of interactive information triggers system scheduling and matching on a big-data-cloud platform. The platform performs and produces outcomes that match; the anesthetic plan is output directly if the platform matching is successful; otherwise, it is output using predetermined parameters with a confirmation or modification interface, followed by marking the modified cases and uploading the actual data in real time to the platform.

**Fig. 3:**
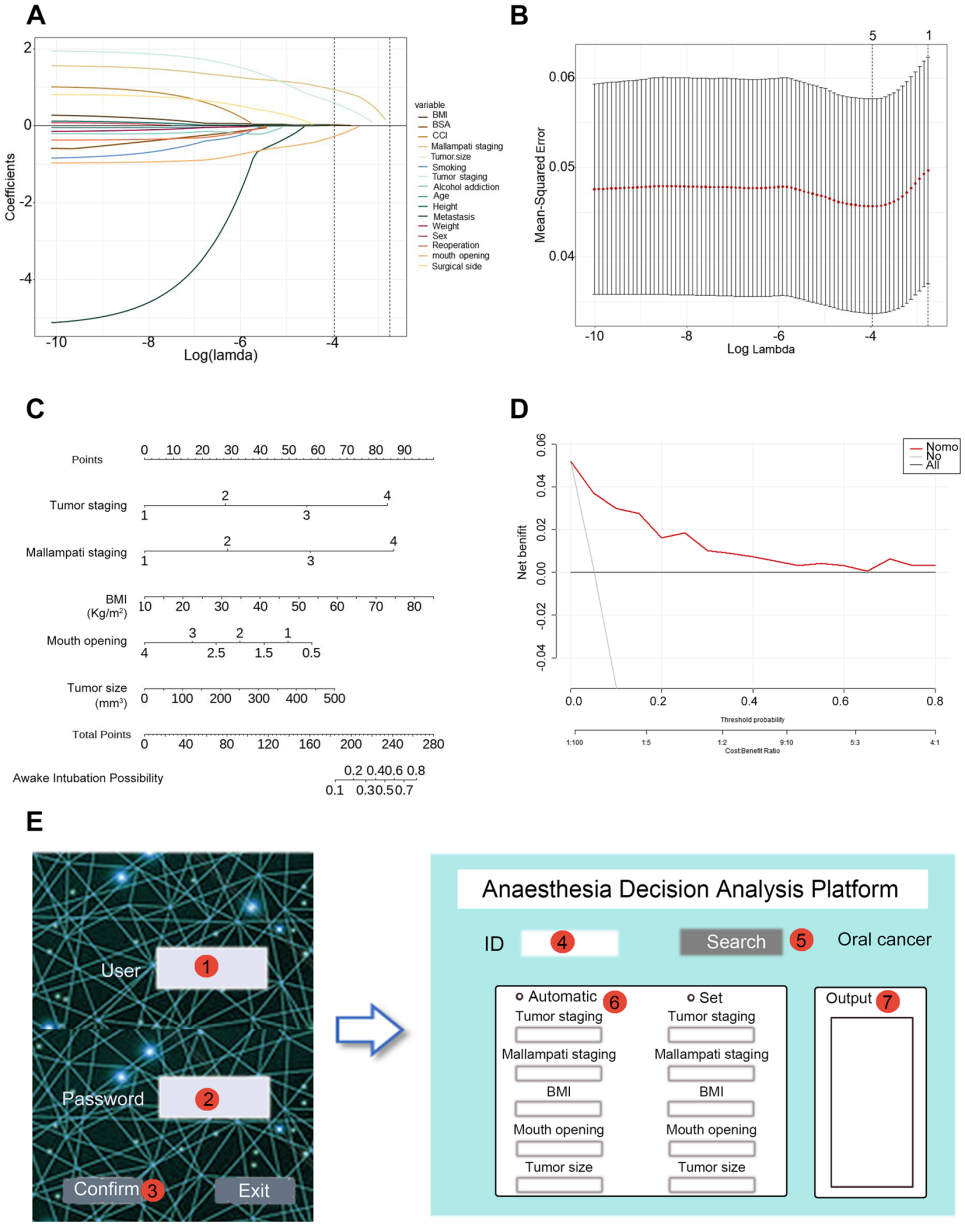
Example of default parameter development and results interpretation in oral cancer patients. **A**, **B** Variable shrinkage and selection using LASSO regression. **C** Nomogram of the model for predicting the wake tracheal intubation. **D** DCA curves were used to evaluate the model. *LASSO* Least absolute shrinkage and selection operator; *DCA* decision curve analysis; *BMI* body mass index; *BSA* body surface area; *CCI* Charlson comorbidity index. **E** A potential user interface presentation. ① User name; ② Password; ③ Log in; ④ Input of registration number; ⑤ Search in Platform; ⑥ Parameter identification; ⑦ Output of anesthesia strategy

The output of the anesthesia strategy is generated by combining various physiological and preoperative data to generate advice, such as the precise identification of the appropriate use of narcotic and auxiliary drugs with the most recent recommendations and dosage schedules, smart alerts, and efficient gas administration. An anesthesiologist may accept or improve the output data. After completing an anesthetic case, the data are returned to the platform for data optimization and updating using Q-learning and Feedback learning algorithms [33]. To ensure quality control, our team involves three or more researchers in charge of data filtering to ensure that patient data are consistent, complete, and accurate. Data processing necessitates consistency and comparability when analyzing the same patient data from many sources. Time series data must have timestamps. Additionally, we sample and evaluate data regularly, anesthesia professionals review any incorrect results, and renew them to the platform following changes. Further, we provide an example of default parameter development, performance of machine-learning, and results’ interpretation in patients with oral cancer to make the platform understandable, because oral lesions significantly influence anesthetic decision-making in clinical practice (Fig. 3, Additional file 1: Fig S1, Additional file 3: Table S2).

### Featured functions of the anesthesiology decision analysis platform

#### Case retrieval

The scientific record retrieval system of the platform modified the previous process of obtaining medical records from various business systems [34]. The platform's system is based on clinical data centers and it can search for medical records that match the indicators from illness data marts using a hospital identification number, a single condition, or several combined diseases. The search results include information such as the anesthetic method and dosage, and expected surgery and resuscitation times.

#### Special case: anesthesia view

The anesthesia views’ special case shows all the medical data generated during the entire process of a specialized case, from the initial diagnosis and surgery to postoperative complications and hospital release. The information created by the case during diagnosis and treatment is displayed fully and accurately using the time axis and presentation dimensions of the type of medical information. The anesthesia view reflects more refined clinical reasoning throughout the perioperative period. Focusing on specialist scientific instances can demonstrate the scientific research value of medical information.

#### Self-service data analysis

Machine learning analysis of big data offers significant advantages for absorbing and evaluating enormous amounts of complex medical data [35]. The learning module of the Anesthesiology Decision Analysis Platform for parameter and model selection is a refinement and summary of traditional learning models that use various algorithms. The training data are used to screen key variables and construct the model, while the validation data are used to verify the results the training cases. Cross-validation and model evaluation indicators (including the F1 score, accuracy, precision, recall, area under the curve, and so on) are used to evaluate the performance of the models [36]. The best-performing models will be selected for further analyses, including feature importance evaluation, identification of key risk factors, and establishment of a comprehensive prediction model. Decision curve analysis and reasonability analysis are used to determine the net clinical benefit and interpretability of model [37].

To ensure that the platform is ready for clinical use as soon as possible, the developers (both anesthesiologists and software engineers) will validate it during the development phase, and then two clinical anesthesiologists provide real-time feedback during the trial process. When a given disease accumulates to a certain level, the platform will analyze the data again, determining the best model, and then testing and validating it once more. The platform applies classical machine-learning algorithms (logistic regression, random forest, extreme gradient boosting [XGBoost], support vector machines [SVM], k-nearest neighbors [KNN], and light gradient boosting machine [LightGBM], among others) to clinical anesthesia data and encapsulates them into a series of machine-learning service options, freeing users from the problems of complex manual operations, simplifying the research and analysis process, and improving scientific research efficiency [38].

Role of the Anesthesiologists in Big Data Platform Development

The widespread use of big data platforms will likely to improve computer-assisted human performance. Clinician–data interactions have previously been shown to improve decision-making [39]. Using big data, anesthesiologists may actively drive cloud-based platform advancements in anesthesia-related medical care, rather than passively waiting for the technology to become useful. First, because a lack of data can limit big-data platform predictions, anesthesiologists should attempt to broaden their involvement in perioperative data registries to ensure that all factors and patients are included. These can contain registers at numerous institutions, regions, and even at national and international levels. As data cleaning and processing techniques improve, registries may boost their utility and the availability of genomic, proteomic, and pathology data. As key stakeholders in adopting cloud-based big data technologies for perioperative decision-making and postoperative resuscitation, anesthesiologists should seek opportunities to collaborate with data scientists to explain how the big data platform can help decision-making with interpretable risk predictions [40, 41].

Furthermore, anesthesiologists can add value to data scientists by sharing their understanding of the relationship between seemingly simple topics, such as physiology, and more complex phenomena, like the dosage of narcotics or postoperative problems. These types of interactions are vital for accurately modeling and predicting clinical events, as well as enhancing the interpretability of cloud-computing platforms. In addition, anesthesiologists are ultimately responsible for making anesthesia decisions for patients and can establish a patient communication framework to relay the data made available by big data platforms and convey the results of complex analyses, such as risk predictions, prognostications, and treatment algorithms, to patients within the anesthesia decision.

## Limitations of big data platforms

A big data platform is not ‘magic bullets’ that answers all questions. In some cases, cloud computing combined with traditional analytical approaches does not improve the results. The use of big data depends on asking the right scientific questions and having adequate data to answer them [42]. Therefore, various limitations or requirements must be addressed before they can be efficiently utilized in clinics.

The types and intricate causal relationships of the accessible data may limit the data output. Systematic biases in clinical data collection can impair the accuracy with which data are recognized or predicted, and this can be especially problematic for neonates and racial minorities because of their long-standing under-representation in clinical trials and patient registry populations. Given the involvement of many anesthesiologists, a platform's decision-making and judgment may be influenced by the diverse anesthetic methods used for the same patient. Additionally, because the platform uses hospital identification, different surgery sites and methods utilized for the same patient may have different anesthesia plans, and the platform's output may be unable to distinguish intelligently and make judgments. In addition, the system may not be able to precisely discern causal linkages in data at the level required for clinical implementation, nor will it provide a clinical interpretation of its studies.

Although big data platforms are convenient, they also pose hazards related to big data management information leakage. One major concern with cloud algorithms is data privacy and security, because they frequently leverage sensitive personal data to generate tailored anesthetic choice suggestions [41, 42]. In an attack, big data value information and the platform's vital data can be manipulated, resulting in cloud-based system failures or serious security issues.

Another crucial clinical concern is responsibility for medical errors [43]. In instances of medical errors, the anesthesiologist may bear the majority of the blame, because clinical tasks either involve or are supervised by an anesthesiologist, although legal experts are still debating whether the developer of the machine-learning tool should bear some blame [44]. The Anesthesia Decision Analysis Platform should thus comply with the medical standard practice guidelines, although standards may change as these technologies become more commonly applied in clinical decision-making.

## Conclusions and future work

In summary, the cloud-based big data platform is expanding its footprint in clinical systems from database analysis to clinical decision-making. In this study, we explored the most recent big data platform achievements in anesthesiology and created a big-data-cloud platform to process anesthesia decision analysis. The database, which can load and store large amounts of data, was created using a distributed cloud architecture. One of the platform's key roles is maintaining many anesthesia decision-making records in the cloud. Users can use the platform's log interface to match efficiently and output anesthetic methods by merging various physiological and preoperative data to provide guidance. The platform also has reinforcement and feedback features for data optimization and updating. Because of the unique nature of the clinical practice, anesthesiologists are well positioned to usher in a new phase of the anesthetic decision-making big data platform, which promises a future optimized for the highest quality anesthetic approach.

### Supplementary Information

**Additional file 1: Fig. S1.** Receiver operating characteristic (ROC) curves of machine-learning models in patients with oral cancer. LR: Logistic regression; KNN: K-nearest neighbours; RF: Random forest; SVM: Support vector machine; XGBoost: Extreme gradient boosting; LightGBM: Light Gradient Boosting Machine; AUC: Area under the curve.

**Additional file 2: Table S1.** The architecture of the Anesthesiology Decision Analysis Platform.

**Additional file 3: Table S2.** Performance summary of machine-learning models in patients with oral cancer.

## Data Availability

Not applicable.
