# Supplementary figures and images for: Anesthesia decision analysis using a cloud-based big data platform
Source: Eur J Med Res. 2024 Mar 25;29:201. doi: 10.1186/s40001-024-01764-0 (PMC10962079; doi:10.1186/s40001-024-01764-0)

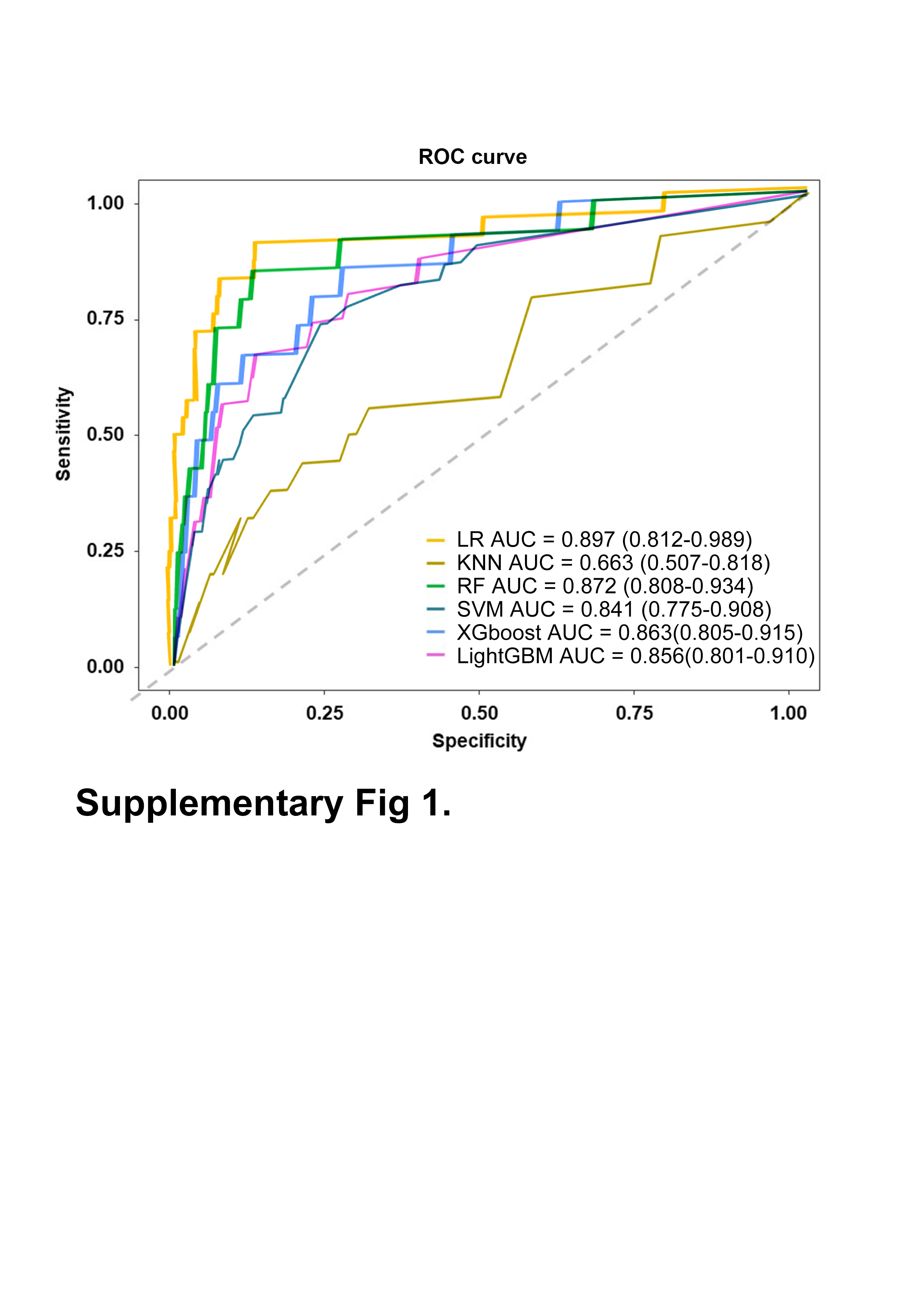

Supplement: Supplementary file 1 — Additional file 1: Fig. S1. Receiver operating characteristic (ROC) curves of machine-learning models in patients with oral cancer. LR: Logistic regression; KNN: K-nearest neighbours; RF: Random forest; SVM: Support vector machine; XGBoost: Extreme gradient boosting; LightGBM: Light Gradient Boosting Machine; AUC: Area under the curve. [file 40001_2024_1764_MOESM1_ESM.tif]
