# Supplementary material for: Anesthesia decision analysis using a cloud-based big data platform
Source: Eur J Med Res. 2024 Mar 25;29:201. doi: 10.1186/s40001-024-01764-0 (PMC10962079; doi:10.1186/s40001-024-01764-0)
Supplement: Supplementary file 2 — Additional file 2: Table S1. The architecture of the Anesthesiology Decision Analysis Platform. [file 40001_2024_1764_MOESM2_ESM.docx]

**Supplementary Table 1.** **The architecture of the Anesthesiology Decision Analysis Platform.**

| **procedure** | **System and Software** |
| --- | --- |
| Raw data acquisition from medical systems | MySQL, HBase of Hadoop, Hadoop Distributed File System [19]  CSV [21], DataX |
| Analysis of intermediate output data | Python [22], Anaconda [23] and R language [24] |
| Clinical data classification into categories | ETL [25], Azkaban System [26] |
| Data Retrieval and Presentation | Presto engine |
| Data Presentation | Apache Echarts [27], DataViz [28] |
| Upgrades & Platform Security | Apache Ranger, Griffin |
