# Supplementary material for: Anesthesia decision analysis using a cloud-based big data platform
Source: Eur J Med Res. 2024 Mar 25;29:201. doi: 10.1186/s40001-024-01764-0 (PMC10962079; doi:10.1186/s40001-024-01764-0)
Supplement: Supplementary file 3 — Additional file 3: Table S2. Performance summary of machine-learning models in patients with oral cancer. [file 40001_2024_1764_MOESM3_ESM.docx]

**Table 3.** **Performance summary of machine learning models in patients with oral cancer**.

| **Models** | **Accuracy (95% CI)** | **Precision (95% CI)** | **Recall (95% CI)** | **F1 (95% CI)** | **AUC (95% CI)** |
| --- | --- | --- | --- | --- | --- |
| LR | 0.926(0.854- 0.969) | 0.656(0.567-0.745) | 0.502 (0.420~0.584) | 0.616 (0.553~0.705) | 0.897 (0.812-0.989) |
| KNN | 0.696 (0.627-0.766) | 0.416 (0.348~0.487) | 0.316 (0.230~0.378) | 0.387(0.317~0.458) | 0.663 (0.507-0.818) |
| RF | 0.883(0.804-0.937) | 0.545(0.472~0.611) | 0.473 (0.401~0.562) | 0.517(0.438~0.600) | 0.872 (0.808-0.934) |
| SVM | 0.867 (0.825-0.906) | 0.578 (0.537~0.618) | 0.462 (0.422-0.501) | 0.495 (0.454-0.535) | 0.841 (0.775-0.908) |
| XGBoost | 0.894 (0.825- 0.944) | 0.582(0.513~0.652) | 0.421(0.351~0.496) | 0.556 (0.485~0.629) | 0.863(0.805-0.915) |
| LightGBM | 0.843 (0.807- 0.874) | 0.572(0.513~0.632) | 0.465(0.394~0.476) | 0.528 (0.490~0.569) | 0.856(0.801-0.910) |
